# Modeling studies for designing transcranial direct current stimulation protocol in Alzheimer's disease

**DOI:** 10.3389/fncom.2014.00072

**Published:** 2014-07-22

**Authors:** Shirin Mahdavi, Fatemeh Yavari, Shahriar Gharibzadeh, Farzad Towhidkhah

**Affiliations:** Department of Biomedical Engineering, Amirkabir University of TechnologyTehran, Iran

**Keywords:** brain stimulation, transcranial direct current stimulation (tDCS), computational modeling, finite element model, human head model, Alzheimer's disease

Transcranial direct current stimulation (tDCS) has been proposed as a technique for brain activity modulation. In this technique, a weak current (usually 1–2 mA) is delivered to scalp through two sponge electrodes. There are two types of tDCS stimulation: cathodal and anodal, which inhibit and facilitate neuronal activity, respectively (Hansen, 2012).

tDCS has been shown to be effective in Alzheimer's disease (AD). Several studies have revealed that tDCS application can improve memory performance in Alzheimer's patients (APs) (Ferrucci et al., 2008; Boggio et al., 2009, 2012). For example, results of a single session tDCS study (Ferrucci et al., 2008) revealed that anodal/cathodal tDCS significantly enhanced/worsened word recognition in AD patients. In another study, application of anodal stimulation over DLPFC of APs has led to recognition memory improvement in a visual memory task (Boggio et al., 2009). These effects seem to be persistent, as in a multi-session tDCS study (Boggio et al., 2012), improvement in patients' visual recognition lasted for 4 weeks.

Current pathway through brain plays a key role in the observed effects. Currently, modeling studies provide the only way for determining the pattern of current flow during tDCS. In recent years, finite element modeling has been suggested as a reliable and helpful tool in clinical therapeutic applications (Bikson et al., 2012).

A critical issue which is required to be considered in modeling studies is the inter-individual anatomical variations. A modeling study has shown the profound role of individual cortical morphology in determination of current flow distribution for healthy people (Datta et al., 2012). Also the impact of pathologic anatomy (skull defects and lesions) on modulation of current flow has been examined in some previous studies (Datta et al., 2010, 2011). Specifically, in AD loss of neuronal structures and synaptic damages result in cortex shrinkage and ventricular enlargement (Frisoni et al., 2010). This changes the volume of CSF- referred as “super highway” for current flow- and therefore can significantly alters current pathway in these patients' head compared to healthy subjects (Bikson et al., 2012). These studies suggest that it is not precise to determine the dosage of applied current only based on healthy human modeling or clinical trial outcomes.

We hypothesize that change in cortical thickness due to brain atrophy has significant effects on current flow pattern. These anatomical alterations may shift the stimulated areas and peak current density location in head. They may even alter the expected results from tDCS application.

We suggest that cortical thickness is required to be considered in modeling studies to obtain more precise pattern of current flow in head and the stimulated brain regions. Specifically, AD affects differently on each patient's brain structure. We suggest developing individualized models based on each patient's MRI data. These models can be used by clinicians to find the optimal electrode montage and current amplitude for each patient.

Using Individual-based models for designing clinical protocols could provide us with better interpretation of the results.

## Conflict of interest statement

The authors declare that the research was conducted in the absence of any commercial or financial relationships that could be construed as a potential conflict of interest.
